# Prognostic Impact of Blood *MN1* Copy Numbers Before Allogeneic Stem Cell Transplantation in Patients With Acute Myeloid Leukemia

**DOI:** 10.1097/HS9.0000000000000167

**Published:** 2019-02-08

**Authors:** Madlen Jentzsch, Marius Bill, Juliane Grimm, Julia Schulz, Stefanie Beinicke, Janine Häntschel, Karoline Goldmann, Wolfram Pönisch, Georg-Nikolaus Franke, Vladan Vucinic, Michael Cross, Gerhard Behre, Thoralf Lange, Dietger Niederwieser, Sebastian Schwind

**Affiliations:** Department of Hematology and Oncology, University of Leipzig, Leipzig, Germany

## Abstract

High expression of the leukemia-associated gene meningioma-1 (*MN1*) is frequently found at diagnosis of acute myeloid leukemia (AML) and associates with adverse outcomes. The presence of measurable residual disease (MRD) in complete remission (CR) indicates high risk of relapse and worse outcome in AML patients. However, the prognostic impact of *MN1* expression levels as MRD marker has not been evaluated. Digital droplet polymerase chain reaction (ddPCR) is a novel technique allowing sensitive and specific absolute gene expression quantification. We retrospectively analyzed 124 AML patients who received allogeneic hematopoietic stem cell transplantation (HSCT) in CR or CR with incomplete peripheral recovery. Absolute *MN1* copy numbers in peripheral blood were assessed prior to HSCT (median 7; range 0–29 days) using ddPCR. High pre-HSCT *MN1*/Abelson murine leukemia viral oncogene homolog 1 gene (*ABL1*) copy numbers associated with a higher cumulative incidence of relapse after HSCT and—in relapsing patients—shorter time to relapse. In multivariable analysis, high pre-HSCT *MN1*/*ABL1* copy numbers remained an independent prognosticator for relapse after HSCT. Patients with the highest pre-HSCT *MN1*/*ABL1* copy numbers also had the highest risk of relapse. *MN1* copy number assessment also added prognostic information to nucleophosmin 1 gene (*NPM1*) mutation- and brain and acute leukemia, cytoplasmic (*BAALC*) and Wilm's tumor gene 1 (*WT1*) expression-based MRD evaluation. Our study demonstrates the feasibility of the novel ddPCR technique for *MN1*/*ABL1* copy number assessment as a marker for MRD. Evaluation of *MN1*/*ABL1* copy numbers allows the identification of patients at high risk of relapse, independently of other diagnostic risk factors and MRD markers.

## Abstract 2

Supplemental Digital Content is available in the text

## Introduction

For optimal and personalized treatment approaches in acute myeloid leukemia (AML), a reliable risk stratification at diagnosis and during disease course is required.^1–3^ Evaluation of measurable residual disease (MRD) during or after therapy may facilitate risk-adapted treatment decisions for individual AML patients.^2–5^ In today's clinical routine, AML MRD evaluation mostly relies on multiparameter flow cytometry (MFC) which is limited due to complex analyses performed in specialized laboratories^6^ and quantitative reverse transcriptase polymerase chain reaction (qRT-PCR) assays. qRT-PCR is largely restricted to patients harboring stable and determinable fusion transcripts or specific, recurrent gene mutations, for example, mutated nucleophosmin 1 gene (*NPM1*).^2,4,7–9^ Next-generation sequencing studies showed AML to be composed of genetically different clones.^1,3,10^ Some subclones may acquire resistance mechanism during disease course and promote relapse molecularly distinct from the AML at diagnosis.^1,10,11^ Thus, the inclusion of gene expression analyses in an MRD marker panel may improve the sensitivity of MRD detection in AML patients. Recently, gene expressions of Wilm's tumor gene 1 (*WT1*)^12,13^ and brain and acute leukemia, cytoplasmic (*BAALC*)^14–16^ were shown to provide informative MRD data in AML remission.

The gene meningioma-1 (*MN1*) was found highly expressed in primitive (CD34-positive) hematopoietic cells and is downregulated during cell differentiation.^17^ Elevated levels were described in acute leukemias of myeloid and lymphoid lineage^17^ and shown to induce proliferation and inhibit myeloid differentiation.^18,19^ At diagnosis, high *MN1* expression was linked to shorter overall survival (OS) and shorter disease-free survival in younger and older AML patients with normal cytogenetics.^17,20,21^ The feasibility of *MN1* expression levels as MRD marker at a defined point in CR has not yet been evaluated. Only one study in 31 AML patients showed that *MN1* levels during disease course parallel disease-specific alterations (ie, *NPM1* mutations and fusion transcripts *CBFB*-*MYH11* and *RUNX1*-*RUNX1T*).^22^ By contrast, low *MN1* expression levels were found in the peripheral blood and bone marrow of healthy individuals. Thus, high bone marrow or blood *MN1* expression might have potential use for MRD monitoring.^22^ Although allogeneic hematopoietic stem cell transplantation (HSCT) has been indicated as the consolidation therapy offering the highest chance of sustained CR in AML patients,^3,23^ detectable MRD prior to HSCT associates with worse outcomes.^8,14,24^ This may be especially true in reduced intensity or nonmyeloablative (NMA) conditioning regimens, which are increasingly used to allow HSCT in older or comorbid individuals.^25–27^ Here, we evaluated the prognostic impact of *MN1*/Abelson murine leukemia viral oncogene homolog 1 gene (*ABL1*) copy numbers prior to NMA-HSCT. We adopted the novel digital droplet PCR (ddPCR) technique that allows absolute copy number quantification without the need of standard curves.^6,28^ The use of peripheral blood enabled a rapid and easily repeatable approach for MRD measurement with high patient convenience. Our study is the first to evaluate the use of *MN1* expression levels as a prognostic factor in CR in a larger patient cohort.

## Results

### *MN1*/*ABL1* copy numbers in AML patients and healthy individuals

**Figure 1:**
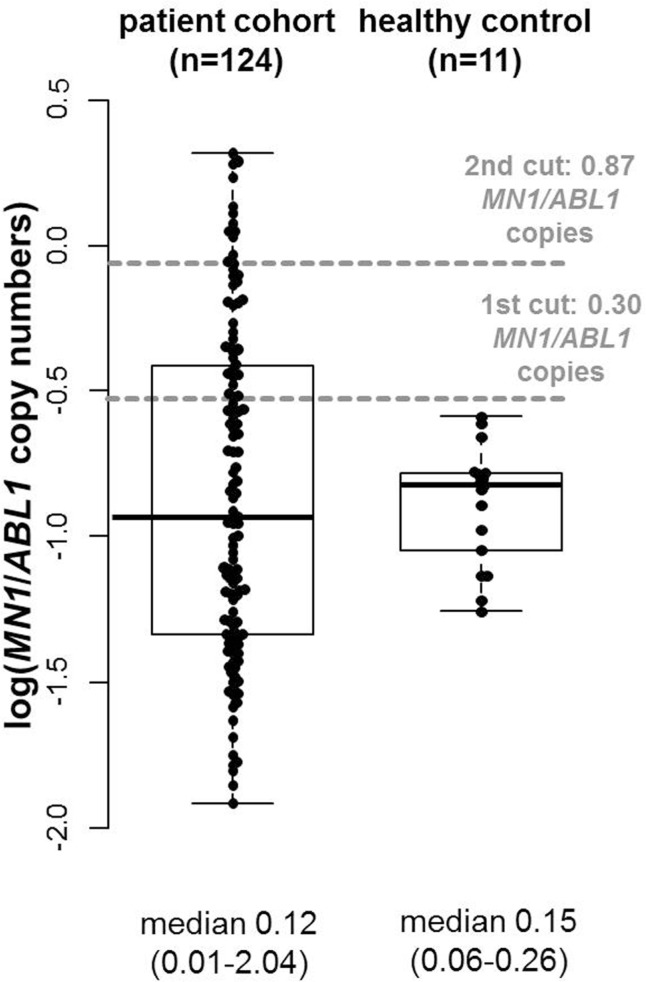
**Comparison of pre-HSCT *MN1/ABL1* copy numbers in AML patients (n = 124) and healthy controls (n = 17).***ABL1* = Abelson murine leukemia viral oncogene homolog 1 gene, AML = acute myeloid leukemia, HSCT = hematopoietic stem cell transplantation, *MN1* = meningioma-1 gene.

In the patient cohort in complete remission (CR) or CR with incomplete peripheral recovery (CRi; median 7, range 0–29 days) prior to allogeneic HSCT, median blood *MN1*/*ABL1* copy numbers were 0.12 (range 0.01–2.04). In the healthy controls, we observed a median blood *MN1*/*ABL1* copy number of 0.15 (range 0.06–0.26). Overall, AML patients in CR or CRi and the healthy control did not differ significantly in *MN1*/*ABL1* copy numbers (*P* = 0.97, Fig. 1) and were evenly matched in sex (*P* = 1) while the healthy control was younger than the AML patients prior to HSCT (*P* = 0.01, Supplementary Table S2, Supplemental Digital Content). For further analyses, a 0.2992 pre-HSCT *MN1*/*ABL1* copy numbers cutoff was used to define patients with high (n = 39, 31%) or low (n = 85, 69%) pre-HSCT *MN1*/*ABL1* copy numbers in peripheral blood.

### Associations of high pre-HSCT *MN1*/*ABL1* copy numbers

Patients with high pre-HSCT *MN1*/*ABL1* copy numbers had a trend for more secondary or treatment-related AML at diagnosis (*P* = 0.07). At diagnosis, patients with high pre-HSCT *MN1*/*ABL1* copy numbers also had a trend for a higher CD34+/CD38− cell burden (*P* = 0.10), a higher white blood count (*P* = 0.02) and no patient with high pre-HSCT *MN1*/*ABL1* copy numbers was *CEBPA* mutated (*P* = 0.05, Table 1). There were no associations of the pre-HSCT *MN1*/*ABL1* copy numbers and other clinical, cytogenetic, molecular, or immunophenotypic characteristics at diagnosis (Table 1, Supplementary Table S1, Supplemental Digital Content). Pre-HSCT *MN1*/*ABL1* copy numbers did also not associate with any tested pre-HSCT characteristics (Supplementary Table S1, Supplemental Digital Content).

**Table 1:**
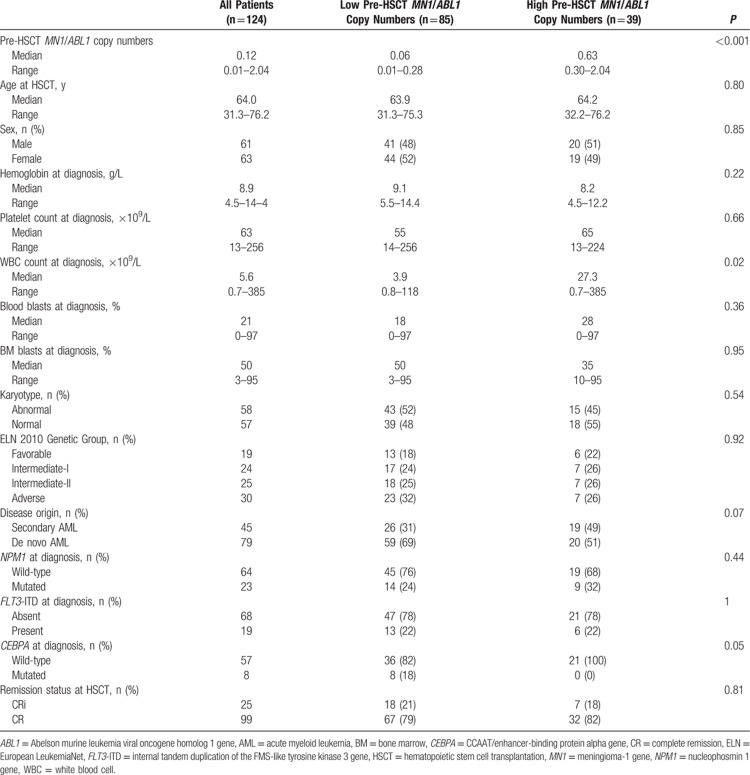
Clinical Characteristics According to Pre-HSCT *MN1*/*ABL1* Copy Numbers (High vs Low, 0.30 Cut), n = 124

### Prognostic impact of pre-HSCT *MN1*/*ABL1* copy numbers

**Figure 2:**
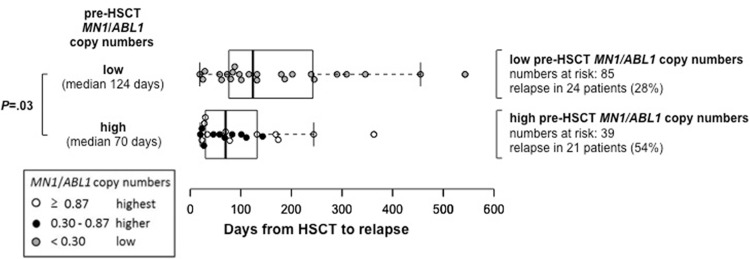
**Time from HSCT to relapse according to high (median 70, range 20–363) or low (median 124, range 19–543) pre-HSCT *MN1/ABL1* copy numbers, 0.30 cut, in patients suffering relapse after HSCT (n = 45).***ABL1* = Abelson murine leukemia viral oncogene homolog 1 gene, HSCT = hematopoietic stem cell transplantation, *MN1* = meningioma-1 gene.

**Figure 3:**
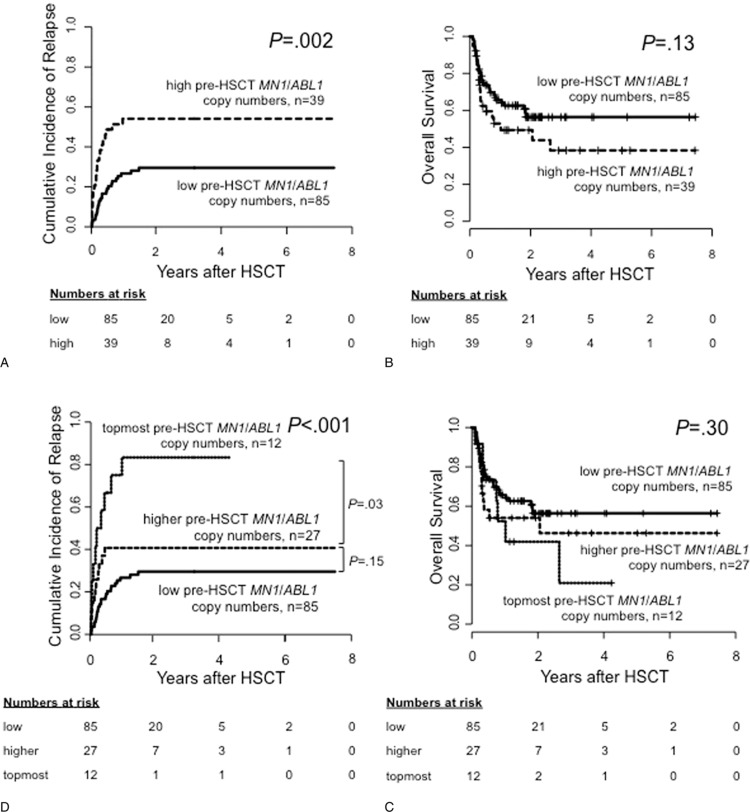
**Outcome according to pre-HSCT MN1/ABL1 copy numbers for the whole cohort (n = 124).** According to high versus low, 0.30 cut, (A) cumulative incidence of relapse and (B) overall survival; and according to the topmost versus higher versus low, 0.87 and 0.30 cut, (C) cumulative incidence of relapse and (D) overall survival. *ABL1* = Abelson murine leukemia viral oncogene homolog 1 gene, HSCT = hematopoietic stem cell transplantation, *MN1* = meningioma-1 gene.

Considering only patients who relapsed after HSCT, patients with high pre-HSCT *MN1*/*ABL1* copy numbers had a shorter time from HSCT to relapse compared with patients with low pre-HSCT *MN1*/*ABL1* copy numbers (median 70, range 20–363 days vs median 124, range 19–543 days, *P* = 0.03, Fig. 2). Patients with high pre-HSCT *MN1*/*ABL1* copy numbers had a significantly higher cumulative incidence of relapse (CIR, *P* = 0.002, Fig. 3A) which—despite a separation of the OS curves—did not translate into a significant shorter OS (*P* = 0.13, Fig. 3B). By contrast, there was no difference in nonrelapse mortality (NRM) between patients with high or low pre-HSCT *MN1*/*ABL1* copy numbers (*P* = 0.28, Supplementary Fig. S1, Supplemental Digital Content). Similar effects on CIR and OS were also observed when we restricted our analyses to patients with normal karyotype (CIR, *P* = 0.005 and OS, *P* = 0.21; Supplementary Fig. S2, Supplemental Digital Content), de novo AML (CIR, *P* = 0.009 and OS, *P* = 0.006; Supplementary Fig. S3, Supplemental Digital Content), excluding patients receiving HSCT in CRi (CIR, *P* = 0.01 and OS, *P* = 0.10; Supplementary Fig. S4, Supplemental Digital Content) and in a landmark analysis of patients surviving longer than 100 days after HSCT (CIR, *P* = 0.02 and OS, *P* = 0.11; Supplementary Fig. S5, Supplemental Digital Content). In multivariable analysis, high pre-HSCT *MN1*/*ABL1* copy numbers retained their prognostic impact on CIR after adjustment for European LeukemiaNet (ELN) 2010 genetic group (Table 2). None of the tested variables were significantly associated with OS in multivariable analysis in this set of patients.

**Table 2:**
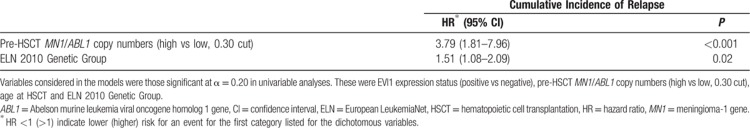
Multivariable Analysis for Patients Receiving HSCT (n = 124)

### CD34 expression at diagnosis and pre-HSCT *MN1*/*ABL1* copy numbers

Although *MN1* was shown to be highly expressed in CD34-positive bone marrow cells,^17^ there are no studies reporting on *MN1* as MRD marker in the context of CD34 expression status. In our study, data on CD34 status at diagnosis were available for 71 patients, 40 patients had CD34-positive and 31 patients had CD34-negative AML. Between patients with high or low pre-HSCT *MN1*/*ABL1* copy numbers, we observed no significant differences of CD34 expression (*P* = 0.35) or CD34-positive disease at diagnosis (*P* = 0.80). Despite low patient numbers, a higher CIR was observed in patients with higher pre-HSCT *MN1*/*ABL1* copy numbers when we restricted our analysis to patients diagnosed with CD34-positive AML (*P* = 0.001, Supplementary Fig. S6A, Supplemental Digital Content). By contrast, there were no significant differences in CIR according to pre-HSCT *MN1*/*ABL1* copy numbers for the 31 patients with CD34-negative AML (*P* = 0.60, Supplementary Fig. S6B, Supplemental Digital Content). In 53 patients, information on the CD34 expression status at diagnosis was not available.

### Prognostic impact of the topmost pre-HSCT *MN1*/*ABL1* copy numbers

To evaluate whether within the group of patients with high pre-HSCT *MN1*/*ABL1* copy numbers, the amount of pre-HSCT *MN1*/*ABL1* copy numbers also impacts on outcome, a second optimal cutoff was applied. Subsequently, the patient cohort was divided into 3 groups according to pre-HSCT *MN1*/*ABL1* copy numbers (0.30 and 0.87 cut): patients with low (n = 85), higher (n = 27), and the topmost (n = 12) pre-HSCT *MN1*/*ABL1* copy numbers. Applying these cutoffs, a stepwise higher CIR was observed with increasing pre-HSCT *MN1*/*ABL1* copy numbers (low vs higher, *P* = 0.15, higher vs topmost, *P* = 0.03, overall *P* < 0.001). However, despite the separation of these curves, again, no significant impact on OS was observed (Fig. 3C and D). Characteristics of patients with higher and the topmost pre-HSCT *MN1*/*ABL1* copy numbers are shown in Supplementary Table S3 (Supplemental Digital Content).

### Correlation of pre-HSCT *MN1*/*ABL1* copy numbers with pre-HSCT *BAALC*/*ABL1* copy number-, *WT1* expression-, and *NPM1* mutation-based MRD

Recently, our group showed the prognostic utility of pre-HSCT *BAALC*/*ABL1* copy numbers and of pre-HSCT *NPM1* for MRD assessment in AML patients undergoing allogeneic HSCT.^8,14^ Correlating *MN1*/*ABL1* and *BAALC*/*ABL1* copy numbers pre-HSCT, patients with high pre-HSCT *MN1*/*ABL1* copy numbers also had higher pre-HSCT *BAALC*/*ABL1* copy numbers (*P* < 0.001). Despite the correlation of pre-HSCT *MN1*/*ABL1* and *BAALC*/*ABL1* copy numbers (Pearson product moment correlation coefficient r = .79), 17 patients (14%) only showed high pre-HSCT expression of 1 of the 2 genes in peripheral blood. Next, we compared the Bayesian information criterion (BIC) for univariable models comprising the pre-HSCT *MN1*/*ABL1* and *BAALC*/*ABL1* copy number information alone or combined (both high vs one or both low) for their predictive value for CIR. Here, the model including the copy number information for both genes combined showed the lowest BIC (Supplementary Table S4, Supplemental Digital Content). This indicates that the evaluation of copy numbers of both genes has higher informative value for MRD assessment than pre-HSCT *MN1*/*ABL1* and *BAALC*/*ABL1* copy number information alone. For outcome analyses according to pre-HSCT *BAALC*/*ABL1* copy numbers and the combination of pre-HSCT *MN1*/*ABL1* and *BAALC*/*ABL1* copy numbers, see Supplementary Information and Supplementary Figure S7 (Supplemental Digital Content).

For 111 of the 124 patients, pre-HSCT *WT1*/*ABL1* expression levels were available. Pre-HSCT *WT1*/*ABL1* expression and *MN1*/*ABL1* copy numbers did not correlate well (Pearson product moment correlation coefficient r = .22, Supplementary Fig. S8, Supplemental Digital Content). However, using the published cutoff of previous work of our institution by Lange et al,^13^ patients with high pre-HSCT *MN1*/*ABL1* copy numbers had significantly higher *WT1*/*ABL1* expression (*P* < 0.001, Supplementary Table S1, Supplemental Digital Content). While patients with high pre-HSCT *WT1*/*ABL1* expression had a significantly higher CIR (Supplementary Fig. S9A, Supplemental Digital Content), pre-HSCT *MN1*/*ABL1* copy number assessment provided additional prognostic information to *WT1*/*ABL1* expression (Supplementary Fig. S9B, Supplemental Digital Content). BIC comparison showed that the model with both pre-HSCT *MN1* and *WT1* expression provided higher informative value than the models with either MRD marker alone (Supplementary Table S5, Supplemental Digital Content).

**Figure 4:**
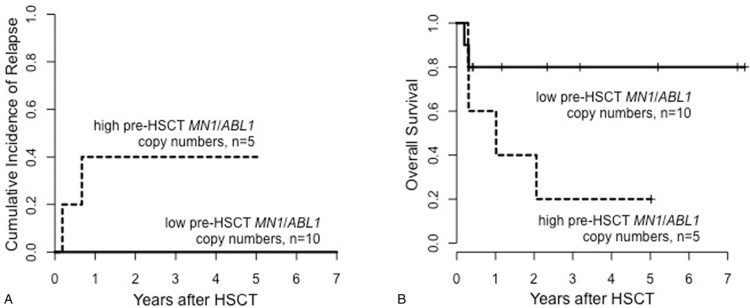
**Outcome according to pre-HSCT *MN1/ABL1* copy numbers, high versus low, 0.30 cut, in mutated NPM1 MRD^neg^ patients (n = 15).** (A) Cumulative incidence of relapse and (B) overall survival. *ABL1* = Abelson murine leukemia viral oncogene homolog 1 gene, HSCT = hematopoietic stem cell transplantation, *MN1* = meningioma-1 gene, MRD = measurable residual disease, *NPM1* = nucleophosmin 1 gene.

For 20 of the 23 *NPM1*-mutated patients, information of pre-HSCT *NPM1* MRD status was available (Supplementary Table S1, Supplemental Digital Content). Four of the 5 *NPM1* MRD positive (MRD^pos^), patients also had high pre-HSCT *MN1*/*ABL1* copy numbers. Two of them relapsed, while 2 patients died of NRM within 100 days after HSCT. One *NPM1* MRD^pos^ patient had low pre-HSCT *MN1*/*ABL1* copy numbers and relapsed. In the 15 mutated *NPM1* MRD negative (MRD^neg^) patients, 5 had high pre-HSCT *MN1*/*ABL1* copy numbers. Of those, 2 patients relapsed, 1 patient died of NRM within 100 days after HSCT, and 2 patients are in continued remission. None of the 10 *NPM1* MRD negative patients with low pre-HSCT *MN1*/*ABL1* copy numbers relapsed. In the 15 mutated *NPM1* MRD^neg^ patients, we observed a clear separation of CIR (Fig. 4A) and OS (Fig. 4B) curves according to pre-HSCT *MN1*/*ABL1* copy numbers, indicating higher CIR and shorter OS for patients with high pre-HSCT *MN1*/*ABL1* copy numbers. In all 5 relapsing patients with information on *MN1*/*ABL1* copy number and *NPM1* MRD status pre-HSCT available, relapse could be predicted prior to HSCT by one (only *NPM1* [n = 1], only *MN1* [n = 2]), or both (n = 2) markers.

## Discussion

Evaluation of MRD during or after AML therapy is of growing importance as it allows dynamic and personalized risk stratification.^2^ Thus, MRD assessment in AML patients is increasingly integrated in clinical trials and daily routine.^3,4,29^ However, AML relapse might be mediated by clones that gained additional mutations or subclones genetically distinct to the AML clone that caused the initial leukemia.^1,3,10^ Consequently, evaluation of more than 1 MRD marker might help to improve risk-adapted treatment.

The transcription factor MN1 was first described in the rare t(12;22)(p13;q11) AML as fusion partner of TEL resulting in a fusion protein with oncogenic potential.^30^*MN1* is overexpressed in AML with inv(16)(p13q22),^18,22,31^ high *EVI1* expression,^31^ and some cases with normal karyotype.^17,22^ In the latter, a strong association of high *MN1* expression at diagnosis with adverse outcomes in younger and older AML patients was described.^17,20,21^ By contrast, low *MN1* expression levels were found in the bone marrow and peripheral blood of healthy individuals.^22^ Assessing *MN1* expression as a potential MRD marker, Carturan et al^22^ demonstrated *MN1* expression to be parallel to simultaneously evaluated fusion gene transcript levels. However, the feasibility of *MN1* copy number measurement for MRD assessment in morphologic CR in a larger AML patient cohort has not been evaluated. In this study, we retrospectively analyzed peripheral blood of AML patients in hematologic CR prior to allogeneic HSCT for consolidation therapy. Using ddPCR, absolute *MN1*/*ABL1* copy numbers were assessed in all patients and a healthy control cohort. The determined optimal cutoff to differentiate between patients with high or low pre-HSCT *MN1*/*ABL1* copy numbers was also higher than the 2-fold standard deviation over the median of the healthy control cohort, allowing a reliable distinction to a physiological *MN1* expression background. While we observed no differences in pre-HSCT clinical characteristics such as numbers of chemotherapy cycles or remission status (Table 1, Supplementary Table S1, Supplemental Digital Content), which matches the literature on other MRD markers,^8,14^ patients with high pre-HSCT *MN1*/*ABL1* copy numbers had a significantly higher risk of relapse compared with patients with low pre-HSCT *MN1*/*ABL1* copy numbers (*P* = 0.002, Fig. 3A). Despite a separation of the OS curves, OS was not significantly different according to pre-HSCT *MN1*/*ABL1* copy number status, likely due to the restricted patient number in our study.

In multivariable analysis, the impact of high pre-HSCT *MN1*/*ABL1* copy numbers on relapse was shown to be independent from other known risk factors in AML (Table 2). We also observed that the time from HSCT to relapse was significantly shorter in relapsing patients with high pre-HSCT *MN1*/*ABL1* copy numbers than in patients with low pre-HSCT *MN1*/*ABL1* copy numbers (*P* = 0.03, Fig. 2). In the group of patients with high pre-HSCT *MN1/ABL1* copy numbers (*n* = 39), we evaluated if increasing *MN1/ABL1* copy numbers also associated with a higher relapse risk. Patients with the topmost pre-HSCT *MN1*/*ABL1* copy numbers (>0.87) also had the highest risk of suffering relapse after HSCT (*P* < 0.001, Fig. 3C): 1 year after HSCT, in patients with the topmost pre-HSCT *MN1*/*ABL1* copy numbers CIR was 71% compared with 41% in patients with higher (0.30–0.87) and only 27% in patients with low (<0.30) pre-HSCT *MN1*/*ABL1* copy numbers. Again, despite no significant differences in OS, a separation of the OS curves was observed according to pre-HSCT *MN1*/*ABL1* copy numbers using both cutoffs (Fig. 3D). We conclude that not only a “higher than normal” *MN1* copy number correlates with a higher relapse risk but that the absolute amount of *MN1*/*ABL1* copy numbers may also provide additional prognostic information. A correlation of higher MRD levels with higher relapse risk has also recently been described for MFC MRD assessment.^32^

Carturan et al^22^ suggested *MN1* as a possible MRD marker with particular benefit in 45% of AML cases lacking other suitable genetic MRD markers. It remains to be investigated for which subset of AML patients *MN1* copy number analysis for MRD detection will be most informative. In our study, subgroup analyses of patients with normal karyotype (Supplementary Fig. S2, Supplemental Digital Content) or de novo disease (Supplementary Fig. S3, Supplemental Digital Content) showed resembling impact on outcome as in the whole cohort. In previous reports, high *MN1* expression at diagnosis was linked to immature AML subtypes with higher CD34 expression.^17,33^ Thus, we investigated the possibility to predict relapse within the patient cohorts with CD34-positive AML (n = 40) and CD34-negative AML (n = 31) at diagnosis. Here, we observed a strong impact of high pre-HSCT *MN1*/*ABL1* copy numbers on CIR in patients with CD34-positive AML (*P* = 0.001, Supplementary Fig. 6A) while no impact was observed in patients with CD34-negative AML (*P* = 0.60, Supplementary Fig. 6B). This suggests that evaluation of pre-HSCT *MN1*/*ABL1* copy numbers might be of higher value in patients with an immature CD34-positive AML phenotype. However, these subanalyses were restricted by limited patient numbers. Furthermore, *NPM1*-mutated AML was enriched in the CD34-negative cohort (53% of patients), which might explain low relapse rates and the missing prognostic impact of pre-HSCT *MN1*/*ABL1* copy numbers in these patients. Larger trials should evaluate for which subgroups of patients *MN1* assessment in CR is of the highest prognostic significance.

As each MRD assay (PCR vs MFC) and marker (fusion gene vs gene mutation vs gene expression) has distinct advantages and disadvantages, combining more than 1 marker for MRD assessment will presumably improve risk stratification.^7,34,35^ Thus, we also evaluated *MN1* MRD results in the context of 3 other MRD markers available for our patient set. Previously, our institution and others were able to show that *BAALC* and *WT1* may function as markers for residual disease in patients after chemotherapy as well as prior to HSCT.^12–16^ As expected, in the here presented cohort, high pre-HSCT *BAALC*/*ABL1* copy numbers also associated with a higher CIR (*P* = 0.007) and a trend for shorter OS (*P* = 0.08, Supplementary Information). When we combined the information of pre-HSCT *BAALC*/*ABL1* and *MN1*/*ABL1* copy numbers (both high vs one or both low, Supplementary Fig. S7C and D, Supplemental Digital Content), our data suggested that evaluation of both genes might be more informative with respect to the risk of relapse after HSCT. Similarly, a high pre-HSCT *WT1*/*ABL1* expression associated with higher CIR (Supplementary Fig. S9A, Supplemental Digital Content). Combining MRD information of *WT1* and *MN1* also provided additional prognostic information: patients with high expression of either of both markers had higher CIR than patients with low expression of both markers but lower CIR than patients with high expression of both markers (overall *P* < 0.001, Supplementary Fig. S9B, Supplemental Digital Content). One of the most established MRD markers in AML is *NPM1* mutations, which are present in approximately 35% of AML patients at diagnosis.^4,8,9,36^ In our cohort, information on pre-HSCT *NPM1* MRD status was available for 20 *NPM1*-mutated patients. In the 15 mutated *NPM1* MRD^neg^ patients pre-HSCT, we observed a clear separation of the CIR and OS curves (Fig. 4) according to pre-HSCT *MN1*/*ABL1* copy numbers. Two of the five relapsing patients were mutated *NPM1* MRD^neg^ prior to HSCT but had high pre-HSCT *MN1*/*ABL1* copy numbers. These patients may have relapsed with an *NPM1*-negative clone. Unfortunately no patient material for further analyses was available for these patients. *MN1* is known to highly correlate with CD34-positive^17,29^ and *NPM1* wild-type AML.^17,20,21^ By contrast, *NPM1* mutations associate with CD34-negative leukemia.^36^ Thus, *MN1* MRD assessment might complement *NPM1* mutation-based MRD assessment.

In AML, MRD assessment prior to consolidating allogeneic HSCT is increasingly performed.^14,29,32,37^ However, in patients with persisting MRD, the question remains as to which treatment approach—for example, additional chemotherapy prior to HSCT, intensification of conditioning regimens or prophylactic donor lymphocyte infusions—would be feasible to improve outcomes and will have to be subject of future prospective clinical trials. These are also needed to evaluate whether patients with high pre-HSCT *MN1*/*ABL1* copy numbers benefit from an allogeneic NMA-HSCT or will have to be lead to alternative treatment options.

Limitations of our study are the retrospective nature and limited patient numbers in the evaluated subgroups. Thus, prospective trials should validate the prognostic use of *MN1* expression as novel and promising MRD marker.

In conclusion, our study is the first to show that assessment of *MN1*/*ABL1* copy numbers is feasible for MRD evaluation in AML patients. Patients with high pre-HSCT *MN1*/*ABL1* copy numbers had a significantly higher CIR and shorter time to relapse, independent of other known genetic and molecular factors at diagnosis or HSCT-related parameters. Patients with the topmost *MN1*/*ABL1* copy numbers had the highest relapse incidence after HSCT, probably due to a higher residual disease burden in these patients. Our data also indicate that *MN1* copy number assessment may have the potential to improve *BAALC*-, *WT1*-, and *NPM1*-based MRD assessment.

## Materials and methods

### Patients and treatment

We retrospectively analyzed 124 adult AML patients who received allogeneic HSCT at the University of Leipzig between September 2002 and December 2015. Median age at HSCT was 64.0 (range 31.3–76.2) years. For all patients, peripheral blood samples at a median of 7 (range 0–29) days prior to HSCT were available. Prior to HSCT, patients received age-dependent chemotherapy protocols (under or over 60 years), further details are given in the Supplementary Information. All patients were consolidated with HSCT in first (53%) or second CR (27%) or CRi (20%). All patients received NMA conditioning consisting of fludarabine 30 mg/m^2^ for 3 days and 2 Gy total body irradiation prior to HSCT^25,38^ followed by infusion of granulocyte colony stimulating factor-mobilized peripheral blood stem cells. Reasons for applying NMA conditioning as opposed to myeloablative conditioning were age over 50 years for patients receiving unrelated HSCT (n = 104), age over 55 years for patients receiving related HSCT (n = 18), previous autologous HSCT (n = 1), or active infection at HSCT (n = 1). Further patients’ characteristics are provided in Table 1 and Supplementary Table S1, Supplemental Digital Content. Written informed consent for participation in these studies was obtained in accordance with the Declaration of Helsinki. Median follow-up for patients alive was 1.8 years.

### Healthy control cohort

Additionally, peripheral blood of a control cohort of 17 healthy volunteers was evaluated for absolute *MN1*/*ABL1* copy numbers. The healthy individuals had a median age of 53.6 (range 32.5–82.0) years; their characteristics are shown in Supplementary Table S2, Supplemental Digital Content. Written informed consent was obtained for all healthy individuals.

### ddPCR assessment of *MN1*/*ABL1* copy numbers

Mononuclear cells were isolated from peripheral blood. RNA was extracted from 1 × 10^7^ cells and processed to complementary DNA as previously described.^35^ Absolute *MN1* copy numbers were assessed using a probe-based ddPCR assay (BioRad, Hercules, CA; Assay ID: dHsaCPE5040386) according to manufacturer's specifications. Absolute *ABL1* copy numbers were assessed as previously described.^14^ ddPCR was performed on a QX100 platform (BioRad), and QuantaSoft software (BioRad) was used for raw data processing. With the droplet generator, each sample was divided into approximately 10,000 to 20,000 partitions (droplets). After PCR amplification the samples were placed into the droplet reader, where each droplet was read as positive or negative for the gene expression by issuing specific fluorescence signals (FAM and HEX). Redistribution according to the Poisson algorithm determined the absolute target copy number in the original sample.

### *MN1*/*ABL1* cutoff definition

Using the R package “OptimalCutpoints”^39^ the optimal cutoff of 0.2992 absolute pre-HSCT *MN1*/*ABL1* copies (high vs low) was determined to differentiate according to their relapse probability. To evaluate whether *MN1*/*ABL1* quantification in patients with very high pre-HSCT *MN1*/*ABL1* copy number allowed the identification of a very high-risk group, a second optimal cutoff of 0.8693 absolute *MN1*/*ABL1* copy number was assessed in these patients and discriminated a cohort with higher (n = 27, 69% of patients with high pre-HSCT *MN1*/*ABL1* copy numbers) or the topmost pre-HSCT *MN1*/*ABL1* copy numbers (n = 12, 31% of patients with high pre-HSCT *MN1*/*ABL1* copy numbers).

### Flow cytometry, cytogenetics, and molecular markers

In patients with pretreatment bone marrow material available, cytogenetic analyses were performed centrally in our institution using standard banding techniques. In cases were no metaphases could be obtained, fluorescence in situ hybridization was used to screen for recurrent abnormalities (ie, del5/5q, del7/7q, trisomy 8, abn11q23, t(8;21), inv(16), and t(15;17) [n = 5]). At diagnosis, the presence of internal tandem duplication in the *FLT3* gene (*FLT3*-ITD), mutations in the *FLT3* tyrosine kinase domain (*FLT3*-TKD) and in the *NPM1* and *CEBPA* genes were determined as previously described.^40^ Patients were grouped according to the ELN 2010 classification in 4 risk groups.^41^ For 71 patients with material available, the bone marrow CD34 and CD38 expression on mononuclear cells at diagnosis was determined as previously described.^42^ Patients were considered CD34-positive when more than 20% of blasts at diagnosis reacted with the CD34 antibody.^43^

### Analysis of other MRD markers

For all patients, pre-HSCT *BAALC*/*ABL1* copy numbers were evaluated by ddPCR as previously described.^14^ In 111 patients, *WT1*/*ABL1* expression levels prior to HSCT were evaluated using quantitative PCR as previously described.^13^ For 20 patients with *NPM1*-mutated AML, pre-HSCT *NPM1* MRD status was evaluated by ddPCR as previously described.^8^ The applicability of all 3 markers for MRD evaluation has been previously published by our institution.^8,13,14^

### Definition of clinical endpoints and statistical analyses

All statistical analyses were performed using the R statistical software platform (version 3.4.3). CIR was calculated from HSCT to morphologic relapse and OS was calculated from HSCT to death from any cause. Associations of the pre-HSCT *MN1*/*ABL1* copy numbers with baseline clinical, demographic, and molecular features were compared using the Kruskal-Wallis test and Fisher exact test for continuous and categorical variables, respectively. For OS, survival estimates were calculated using the Kaplan-Meier method and groups were compared with the log-rank test. CIR was calculated considering the competing risk NRM using the Fine and Gray model.

## Acknowledgments

The authors thank Christel Müller, Daniela Bretschneider, Evelin Hennig, Dr Sabine Leiblein, Martina Pleß, Ulrike Bergmann, Janet Bogardt, Annette Jilo, and Dagmar Cron for their help in determining cytogenetic, morphologic, and immunological analyses; Christine Günther, Scarlett Schwabe, Ines Kovacs, and Kathrin Wildenberger for their help in sample processing and Dr Max Hubmann and Prof Ralf Burckhardt for their help in *WT1* expression analysis.

## Supplementary Material

Supplemental Digital Content
