# Supplementary material for: Prognostic Impact of Blood MN1 Copy Numbers Before Allogeneic Stem Cell Transplantation in Patients With Acute Myeloid Leukemia
Source: Hemasphere. 2019 Feb 8;3(1):e167. doi: 10.1097/HS9.0000000000000167 (PMC6745933; doi:10.1097/HS9.0000000000000167)
Supplement: Supplemental Digital Content [file hs9-3-e167-s001.docx]

**Prognostic impact of blood *MN1* copy numbers before allogeneic stem cell transplantation in patients with acute myeloid leukemia**

**Abstract**

High expression of the leukemia-associated gene meningioma-1 (*MN1*) is frequently found at diagnosis of acute myeloid leukemia (AML) and associates with adverse outcomes. The presence of measurable residual disease (MRD) in complete remission (CR) indicates high risk of relapse and worse outcome in AML patients. However, the prognostic impact of *MN1* expression levels as MRD marker has not been evaluated. Digital droplet PCR (ddPCR) is a novel technique allowing sensitive and specific absolute gene expression quantification. We retrospectively analyzed 124 AML patients who received allogeneic hematopoietic stem cell transplantation (HSCT) in CR or CR with incomplete peripheral recovery. Absolute *MN1* copy numbers in peripheral blood were assessed prior to HSCT (median 7; range 0-29 days) using ddPCR. High pre-HSCT *MN1*/*ABL1* copy numbers associated with a higher cumulative incidence of relapse after HSCT and - in relapsing patients - shorter time to relapse. In multivariable analysis, high pre-HSCT *MN1*/*ABL1* copy numbers remained an independent prognosticator for relapse after HSCT. Patients with the highest pre-HSCT *MN1*/*ABL1* copy numbers also had the highest risk of relapse. *MN1* copy number assessment also added prognostic information to *NPM1* mutation- and *BAALC* and *WT1* expression-based MRD evaluation. Our study demonstrates the feasibility of the novel ddPCR technique for *MN1*/*ABL1* copy number assessment as a marker for MRD. Evaluation of *MN1*/*ABL1* copy numbers allows the identification of patients at high risk of relapse, independently of other diagnostic risk factors and MRD markers.

**Introduction**

For optimal and personalized treatment approaches in acute myeloid leukemia (AML) a reliable risk stratification at diagnosis and during disease course is required.^1-3^ Evaluation of measurable residual disease (MRD) during or after therapy may facilitate risk-adapted treatment decisions for individual AML patients.^2-5^ In today’s clinical routine, AML MRD evaluation mostly relies on multiparameter flow cytometry (MFC) which is limited due to complex analyses performed in specialized laboratories^6^ and quantitative reverse transcriptase polymerase chain reaction (qRT-PCR) assays. qRT-PCR is largely restricted to patients harboring stable and determinable fusion transcripts or specific, recurrent gene mutations, *e.g.* mutated *NPM1*.^2,4,7-9^ Next generation sequencing studies showed AML to be composed of genetically different clones.^1,3,10^ Some subclones may acquire resistance mechanism during disease course and promote relapse molecularly distinct from the AML at diagnosis.^1,10,11^ Thus, the inclusion of gene expression analyses in a MRD marker panel may improve the sensitivity of MRD detection in AML patients. Today, gene expressions of Wilm’s tumor gene 1 (*WT1*)^12,13^ and brain and acute leukemia, cytoplasmic (*BAALC*)^14-16^ were shown to provide informative MRD data in AML remission.

The gene meningioma-1 (*MN1*) was found highly expressed in primitive (CD34-positive) hematopoietic cells and is downregulated during cell differentiation.^17^ Elevated levels were described in acute leukemias of myeloid and lymphoid lineage^17^ and shown to induce proliferation and inhibit myeloid differentiation.^18,19^ At diagnosis, high *MN1* expression was linked to shorter overall survival (OS) and shorter disease free survival in younger and older AML patients with normal cytogenetics.^17,20,21^ The feasibility of *MN1* expression levels as MRD marker at a defined point in CR has not yet been evaluated. Only one study in 31 AML patients showed that *MN1* levels during disease course parallel disease-specific alterations (*i.e.* *NPM1* mutations and fusion transcripts *CBFB*-*MYH11* and *RUNX1*-*AML1*).^22^ In contrast, low *MN1* expression levels were found in the peripheral blood and bone marrow of healthy individuals. Thus, high bone marrow or blood *MN1* expression might have potential use for MRD monitoring.^22^ Although allogeneic hematopoietic stem cell transplantation (HSCT) has been indicated as the consolidation therapy offering the highest chance of sustained CR in AML patients,^3,23^ detectable MRD prior to HSCT associates with worse outcomes.^8,14,24^  This may be especially true in reduced intensity or non-myeloablative (NMA) conditioning regimens which are increasingly used to allow HSCT in older or comorbid individuals.^25-27^ Here, we evaluated the prognostic impact of *MN1*/*ABL1* copy numbers prior to NMA-HSCT. We adopted the novel digital droplet PCR (ddPCR) technique that allows absolute copy number quantification without the need of standard curves.^6,28^ The use of peripheral blood enabled a rapid and easily repeatable approach for MRD measurement with high patient convenience. Our study is the first to evaluate the use of *MN1* expression levels as a prognostic factor in CR in a larger patient cohort.

**Results**

***MN1/ABL1 copy numbers in AML patients and healthy individuals***

In the patient cohort in complete remission (CR) or CR with incomplete peripheral recovery (CRi; median 7, range 0-29 days) prior to allogeneic HSCT, median blood *MN1*/*ABL1* copy numbers were 0.12 (range 0.01-2.04). In the healthy controls, we observed a median blood *MN1*/*ABL1* copy number of 0.15 (range 0.06-0.26). Overall, AML patients in CR or CRi and the healthy control did not differ significantly in *MN1*/*ABL1* copy numbers (*P*=.97, Figure 1) and were evenly matched in sex (*P*=1) while the healthy control was younger than the AML patients prior to HSCT (*P*=.01, Supplementary Table S2, Supplemental Digital Content, http://links.lww.com/HS/A20). For further analyses, a 0.2992 pre-HSCT *MN1*/*ABL1* copy numbers cut-off was used to define patients with high (n=39, 31%) or low (n=85, 69%) pre-HSCT *MN1*/*ABL1* copy numbers in peripheral blood.

***Associations of high pre-HSCT MN1/ABL1 copy numbers***

Patients with high pre-HSCT *MN1*/*ABL1* copy numbers had a trend for more secondary or treatment related AML at diagnosis (*P*=.07). At diagnosis, patients with high pre-HSCT *MN1*/*ABL1* copy numbers also had a trend for a higher CD34+/CD38- cell burden (*P*=.10), a higher white blood count (*P*=.02) and no patient with high pre-HSCT *MN1*/*ABL1* copy numbers was *CEBPA* mutated (*P*=.05, Table 1). There were no associations of the pre-HSCT *MN1*/*ABL1* copy numbers and other clinical, cytogenetic, molecular or immunophenotypic characteristics at diagnosis (Table 1, Supplementary Table S1, Supplemental Digital Content, http://links.lww.com/HS/A20). Pre-HSCT *MN1*/*ABL1* copy numbers did also not associate with any tested pre-HSCT characteristics (Supplementary Table S1, Supplemental Digital Content, http://links.lww.com/HS/A20).

***Prognostic impact of pre-HSCT MN1/ABL1 copy numbers***

Considering only patients who relapsed after HSCT, patients with high pre-HSCT *MN1*/*ABL1* copy numbers had a shorter time from HSCT to relapse compared to patients with low pre-HSCT *MN1*/*ABL1* copy numbers (median 70, range 20-363 days *vs*. median 124, range 19-543 days, *P*=.03, Figure 2). Patients with high pre-HSCT *MN1*/*ABL1* copy numbers had a significantly higher cumulative incidence of relapse (CIR, *P*=.002, Figure 3A) which - despite a separation of the OS curves - did not translate into a significant shorter overall survival (OS, *P*=.13, Figure 3B). In contrast, there was no difference in non-relapse mortality (NRM) between patients with high or low pre-HSCT *MN1*/*ABL1* copy numbers (*P*=.28, Supplementary Figure S1, Supplemental Digital Content, http://links.lww.com/HS/A20). Similar effects on CIR and OS were also observed when we restricted our analyses to patients with normal karyotype (CIR, *P*=.005 and OS, *P*=.21; Supplementary Figures S2, Supplemental Digital Content, http://links.lww.com/HS/A20), *de novo* AML (CIR, *P*=.009 and OS, *P*=.006; Supplementary Figures S3, Supplemental Digital Content, http://links.lww.com/HS/A20), excluding patients receiving HSCT in CRi (CIR, *P*=.01 and OS, *P*=.10; Supplementary Figures S4, Supplemental Digital Content, http://links.lww.com/HS/A20) and in a landmark analysis of patients surviving longer than 100 days after HSCT (CIR, *P*=.02 and OS, *P*=.11; Supplementary Figures S5, Supplemental Digital Content, http://links.lww.com/HS/A20). In multivariable analysis, high pre-HSCT *MN1*/*ABL1* copy numbers retained their prognostic impact on CIR after adjustment for European LeukemiaNet (ELN) 2010 genetic group (Table 2). None of the tested variables were significantly associated with OS in multivariable analysis in this set of patients.

***CD34 expression at diagnosis and pre-HSCT MN1/ABL1 copy numbers***

Although *MN1* was shown to be highly expressed in CD34-positive bone marrow cells,^17^ there are no studies reporting on *MN1* as MRD marker in the context of CD34 expression status. In our study, data on CD34-status at diagnosis was available for 71 patients, 40 patients had CD34-positive and 31 patients had CD34-negative AML. Between patients with high or low pre-HSCT *MN1*/*ABL1* copy numbers we observed no significant differences of CD34 expression (*P*=.35) or CD34-positive disease at diagnosis (*P*=.80). Despite low patient numbers, a higher CIR was observed in patients with higher pre-HSCT *MN1*/*ABL* copy numbers when we restricted our analysis to patients diagnosed with CD34-positive AML (*P*=.001, Supplementary Figure S6A, Supplemental Digital Content, http://links.lww.com/HS/A20). In contrast, there were no significant differences in CIR according to pre-HSCT *MN1*/*ABL1* copy numbers for the 31 patients with CD34-negative AML (*P*=.60, Supplementary Figure S6B, Supplemental Digital Content, http://links.lww.com/HS/A20). In 53 patients, information on the CD34 expression status at diagnosis was not available.

***Prognostic impact of the topmost pre-HSCT MN1/ABL1 copy numbers***

To evaluate whether within the group of patients with high pre-HSCT *MN1*/*ABL1* copy numbers the amount of pre-HSCT *MN1*/*ABL1* copy numbers also impacts on outcome, a second optimal cut-off was applied. Subsequently, the patient cohort was divided into 3 groups according to pre-HSCT *MN1*/*ABL1* copy numbers (0.30 and 0.87 cut): patients with low (n=85), higher (n=27) and the topmost (n=12) pre-HSCT *MN1*/*ABL1* copy numbers. Applying these cut-offs, a stepwise higher CIR was observed with increasing pre-HSCT *MN1*/*ABL1* copy numbers (low *vs*. higher, *P*=.15, higher *vs*. topmost, *P*=.03, overall *P*<.001). However, despite the separation of these curves, again, no significant impact on OS was observed (Figures 3 C,D). Characteristics of patients with higher and the topmost pre-HSCT *MN1*/*ABL1* copy numbers are shown in Supplementary Table S3 (Supplemental Digital Content, http://links.lww.com/HS/A20).

***Correlation of pre-HSCT MN1/ABL1 copy numbers with pre-HSCT BAALC/ABL1 copy number-, WT1 expression- and NPM1 mutation-based MRD***

Recently, our group showed the prognostic utility of pre-HSCT *BAALC*/*ABL1* copy numbers and of pre-HSCT *NPM1* for MRD assessment in AML patients undergoing allogeneic HSCT.^8,14^ Correlating *MN1*/*ABL1* and *BAALC*/*ABL1* copy numbers pre-HSCT, patients with high pre-HSCT *MN1*/*ABL1* copy numbers also had higher pre-HSCT *BAALC*/*ABL1* copy numbers (*P*<.001). Despite the correlation of pre-HSCT *MN1*/*ABL1* and *BAALC*/*ABL1* copy numbers (Pearson's product moment correlation coefficient r=.79), 17 patients (14%) only showed high pre-HSCT expression of one of the two genes in peripheral blood. Next, we compared the Bayesian information criterion (BIC) for univariable models comprising the pre-HSCT *MN1*/*ABL1* and *BAALC*/*ABL1* copy number information alone or combined (both high *vs.* one or both low) for their predictive value for CIR. Here, the model including the copy number information for both genes combined showed the lowest BIC (Supplementary Table S4, Supplemental Digital Content, http://links.lww.com/HS/A20). This indicates that the evaluation of copy numbers of both genes has higher informative value for MRD assessment than pre-HSCT *MN1*/*ABL1* and *BAALC*/*ABL1* copy number information alone. For outcome analyses according to pre-HSCT *BAALC*/*ABL1* copy numbers and the combination of pre-HSCT *MN1*/*ABL1* and *BAALC*/*ABL1* copy numbers, see Supplementary Information and Supplementary Figure S7 (Supplemental Digital Content, http://links.lww.com/HS/A20).

For 111 of the 124 patients, pre-HSCT *WT1/ABL1* expression levels were available. Pre-HSCT *WT1/ABL1* expression and *MN1/ABL1* copy numbers did not correlate well (Pearson's product moment correlation coefficient r=.22, Supplementary Figure S8, Supplemental Digital Content, http://links.lww.com/HS/A20). However, using the published cut-off of previous work of our institution by Lange *et al.*^13^ patients with high pre-HSCT *MN1*/*ABL1* copy numbers had significantly higher *WT1*/*ABL1* expression (*P*<.001, Supplementary Table S1, Supplemental Digital Content, http://links.lww.com/HS/A20). While patients with high pre-HSCT *WT1*/*ABL1* expression had a significantly higher CIR (Supplementary Figure S9A, Supplemental Digital Content, http://links.lww.com/HS/A20), pre-HSCT *MN1*/*ABL1* copy number assessment provided additional prognostic information to *WT1*/*ABL1* expression (Supplementary Figure S9B, Supplemental Digital Content, http://links.lww.com/HS/A20). BIC comparison showed that the model with both pre-HSCT *MN1* and *WT1* expression provided higher informative value than the models with either MRD marker alone (Supplementary Table S5, Supplemental Digital Content, http://links.lww.com/HS/A20).

For 20 of the 23 *NPM1* mutated patients information of pre-HSCT *NPM1* MRD status was available (Supplementary Table S1, Supplemental Digital Content, http://links.lww.com/HS/A20). Four of the five *NPM1* MRD positive (MRD^pos^) patients also had high pre-HSCT *MN1*/*ABL1* copy numbers. Two of them relapsed, while two patients died of NRM within 100 days after HSCT. One *NPM1* MRD^pos^ patient had low pre-HSCT *MN1*/*ABL1* copy numbers and relapsed. In the 15 mutated *NPM1* MRD negative (MRD^neg^) patients, five had high pre-HSCT *MN1*/*ABL1* copy numbers. Of those, two patients relapsed, one patient died of NRM within 100 days after HSCT and two patients are in continued remission. None of the ten *NPM1* MRD negative patients with low pre-HSCT *MN1*/*ABL1* copy numbers relapsed. In the 15 mutated *NPM1* MRD^neg^ patients, we observed a clear separation of CIR (Figure 4A) and OS (Figure 4B) curves according to pre-HSCT *MN1*/*ABL1* copy numbers, indicating higher CIR and shorter OS for patients with high pre-HSCT *MN1*/*ABL1* copy numbers. In all five relapsing patients with information on *MN1*/*ABL1* copy number and *NPM1* MRD status pre-HSCT available, relapse could be predicted prior to HSCT by one (only *NPM1* [n=1], only *MN1* [n=2]) or both (n=2) markers.

**Discussion**

Evaluation of MRD during or after AML therapy is of growing importance as it allows dynamic and personalized risk stratification.^2^ Thus, MRD assessment in AML patients is increasingly integrated in clinical trials and daily routine.^3,4,29^ However, AML relapse might be mediated by clones that gained additional mutations or subclones genetically distinct to the AML clone that caused the initial leukemia.^1,3,10^ Consequently, evaluation of more than one MRD marker might help to improve risk-adapted treatment.

The transcription factor MN1 was first described in the rare t(12;22)(p13;q11) AML as fusion partner of TEL resulting in a fusion protein with oncogenic potential.^30^ *MN1* is overexpressed in AML with inv(16)(p13q22),^18,22,31^ high *EVI1* expression^31^ and some cases with normal karyotype.^17,22^ In the latter, a strong association of high *MN1* expression at diagnosis with adverse outcomes in younger and older AML patients was described.^17,20,21^ In contrast, low *MN1* expression levels were found in the bone marrow and peripheral blood of healthy individuals.^22^ Assessing *MN1* expression as a potential MRD marker, Carturan *et al*.^22^ demonstrated *MN1* expression to be parallel to simultaneously evaluated fusion gene transcript levels. However, the feasibility of *MN1* copy number measurement for MRD assessment in morphologic CR in a larger AML patient cohort has not been evaluated. In this study we retrospectively analyzed peripheral blood of AML patients in hematologic CR prior to allogeneic HSCT for consolidation therapy. Using ddPCR, absolute *MN1*/*ABL1* copy numbers were assessed in all patients and a healthy control cohort. The determined optimal cut-off to differentiate between patients with high or low pre-HSCT *MN1*/*ABL1* copy numbers was also higher than the two-fold standard deviation over the median of the healthy control cohort, allowing a reliable distinction to a physiological *MN1* expression background. While we observed no differences in pre-HSCT clinical characteristics such as numbers of chemotherapy cycles or remission status (Table 1, Supplementary Table S1) which matches the literature on other MRD markers,^8,14^ patients with high pre-HSCT *MN1*/*ABL1* copy numbers had a significantly higher risk of relapse compared to patients with low pre-HSCT *MN1*/*ABL1* copy numbers (*P*=.002, Figure 3A). Despite a separation of the OS curves OS was not significantly different according to pre-HSCT *MN1*/*ABL1* copy number status, likely due to the restricted patient number in our study.

In multivariable analysis, the impact of high pre-HSCT *MN1*/*ABL1* copy numbers on relapse was shown to be independent from other known risk factors in AML (Table 2). We also observed that the time from HSCT to relapse was significantly shorter in relapsing patients with high pre-HSCT *MN1*/*ABL1* copy numbers than in patients with low pre-HSCT *MN1*/*ABL1* copy numbers (*P*=.03, Figure 2). In patients with high pre-HSCT *MN1*/*ABL1* copy numbers we evaluated if increasing *MN1*/*ABL1* copy numbers also associated with a higher relapse risk. Patients with the topmost pre-HSCT *MN1*/*ABL1* copy numbers (>0.87) also had the highest risk of suffering relapse after HSCT (*P*<.001, Figure 3C): one year after HSCT, in patients with the topmost pre-HSCT *MN1*/*ABL1* copy numbers CIR was 71% compared to 41% in patients with higher (0.30 – 0.87) and only 27% in patients with low (<0.30) pre-HSCT *MN1*/*ABL1* copy numbers. Again, despite no significant differences in OS, a separation of the OS curves was observed according to pre-HSCT *MN1*/*ABL1* copy numbers using both cut-offs (Figure 3D). We conclude that not only a “higher than normal” *MN1* copy number correlates with a higher relapse risk but that the absolute amount of *MN1*/*ABL1* copy numbers may also provide additional prognostic information. A correlation of higher MRD levels with higher relapse risk have also recently been described for MFC MRD assessment.^32^

Carturan *et al*.^22^ suggested *MN1* as a possible MRD marker with particular benefit in 45% of AML cases lacking other suitable genetic MRD markers. It remains to be investigated for which subset of AML patients *MN1* copy number analysis for MRD detection will be most informative. In our study, subgroup analyses of patients with normal karyotype (Supplementary Figures S2) or *de novo* disease (Supplementary Figure S3) showed resembling impact on outcome as in the whole cohort. In previous reports high *MN1* expression at diagnosis was linked to immature AML subtypes with higher CD34 expression.^17,33^ Thus, we investigated the possibility to predict relapse within the patient cohorts with CD34-positive AML (n=40) and CD34-negative AML (n=31) at diagnosis. Here, we observed a strong impact of high pre-HSCT *MN1*/*ABL1* copy numbers on CIR in patients with CD34-positive AML (*P*=.001, Supplementary Figure 6A) while no impact was observed in patients with CD34-negative AML (*P*=.60, Supplementary Figure 6B). This suggests that evaluation of pre-HSCT *MN1*/*ABL1* copy numbers might be of higher value in patients with an immature CD34-positive AML phenotype. However, these subanalyses were restricted by limited patient numbers. Furthermore, *NPM1*-mutated AML was enriched in the CD34-negative cohort (53% of patients), which might explain low relapse rates and the missing prognostic impact of pre-HSCT *MN1*/*ABL1* copy numbers in these patients. Larger trials should evaluate for which subgroups of patients *MN1* assessment in CR is of the highest prognostic significance.

As each MRD assay (PCR *vs*. MFC) and marker (fusion gene *vs*. gene mutation *vs*. gene expression) has distinct advantages and disadvantages, combining more than one marker for MRD assessment will presumably improve risk stratification.^7,38,34^ Thus, we also evaluated *MN1* MRD results in the context of three other MRD markers available for our patient set. Previously, our institution and others were able to show that *BAALC* and *WT1* may function as markers for residual disease in patients after chemotherapy as well as prior to HSCT.^12-16^ As expected, in the here presented cohort, high pre-HSCT *BAALC*/*ABL1* copy numbers also associated with a higher CIR (*P*=.007) and a trend for shorter OS (*P*=.08, Supplementary Information). When we combined the information of pre-HSCT *BAALC*/*ABL1* and *MN1*/*ABL1* copy numbers (both high *vs*. one or both low, Supplementary Figure S7C,D), our data suggested that evaluation of both genes might be more informative with respect to the risk of relapse after HSCT. Similarly, a high pre-HSCT *WT1*/*ABL1* expression associated with higher CIR (Supplementary Figure S9A). Combining MRD information of *WT1* and *MN1* also provided additional prognostic information: patients with high expression of either of both markers had higher CIR than patients with low expression of both markers but lower CIR than patients with high expression of both markers (overall *P*<.001, Supplementary Figure S9B). One of the most established MRD markers in AML are *NPM1* mutations which are present in approximately 35% of AML patients at diagnosis.^4,8,9,35^ In our cohort, information on pre-HSCT *NPM1* MRD status was available for 20 *NPM1* mutated patients. In the 15 mutated *NPM1* MRD^neg^ patients pre-HSCT, we observed a clear separation of the CIR and OS curves (Figure 4) according to pre-HSCT *MN1*/*ABL1* copy numbers. Two of the five relapsing patients were mutated *NPM1* MRD^neg^ prior to HSCT but had high pre-HSCT *MN1*/*ABL1* copy numbers. These patients may have relapsed with a *NPM1*-negative clone. Unfortunately no patient material for further analyses was available for these patients. *MN1* is known to highly correlate with CD34-positive^17,29^ and *NPM1* wild type AML.^17,20,21^ In contrast, *NPM1* mutations associate with CD34-negative leukemia.^35^ Thus, *MN1* MRD assessment might complement *NPM1* mutation based MRD assessment.

In AML MRD assessment prior to consolidating allogeneic HSCT is increasingly performed.^14,29,32,36^ However, in patients with persisting MRD the question remains as to which treatment approach - *e.g.* additional chemotherapy prior to HSCT, intensification of conditioning regimens or prophylactic donor lymphocyte infusions - would be feasible to improve outcomes and will have to be subject of future prospective clinical trials. These are also needed to evaluate whether patients with high pre-HSCT *MN1*/*ABL1* copy numbers benefit from an allogeneic NMA-HSCT or will have to be lead to alternative treatment options.

Limitations of our study are the retrospective nature and limited patient numbers in the evaluated subgroups. Thus, prospective trials should validate the prognostic use of *MN1* expression as novel and promising MRD marker.

In conclusion, our study is the first to show that assessment of *MN1*/*ABL1* copy numbers is feasible for MRD evaluation in AML patients. Patients with high pre-HSCT *MN1*/*ABL1* copy numbers had a significantly higher CIR and shorter time to relapse, independent of other known genetic and molecular factors at diagnosis or HSCT-related parameters. Patients with the topmost *MN1*/*ABL1* copy numbers had the highest relapse incidence after HSCT, probably due to a higher residual disease burden in these patients. Our data also indicates that *MN1* copy number assessment may have the potential to improve *BAALC*-, *WT1*- and *NPM1*-based MRD assessment.

**Material and methods**

***Patients and treatment***

We retrospectively analyzed 124 adult AML patients who received allogeneic HSCT at the University of Leipzig between September 2002 and December 2015. Median age at HSCT was 64.0 (range 31.3-76.2) years. For all patients, peripheral blood samples at a median of 7 (range 0-29) days prior to HSCT were available. Prior to HSCT, patients received age-dependent chemotherapy protocols (under or over 60 years), further details are given in the Supplementary Information. All patients were consolidated with HSCT in first (53%) or second CR (27%) or CRi (20%). All patients received NMA conditioning consisting of fludarabine 30 mg/m^2^ for three days and 2 Gy total body irradiation prior to HSCT^25,37^ followed by infusion of granulocyte colony stimulating factor (G-CSF)-mobilized peripheral blood stem cells. Reasons for applying NMA conditioning as opposed to myeloablative conditioning were age over 50 years for patients receiving unrelated HSCT (n=104), age over 55 years for patients receiving related HSCT (n=18), previous autologous HSCT (n=1) or active infection at HSCT (n=1). Further patients’ characteristics are provided in Table 1 and Supplementary Table S1. Written informed consent for participation in these studies was obtained in accordance with the Declaration of Helsinki. Median follow up for patients alive was 1.8 years.

***Healthy control cohort***

Additionally, peripheral blood of a control cohort of 17 healthy volunteers was evaluated for absolute *MN1*/*ABL1* copy numbers. The healthy individuals had a median age of 53.6 (range 32.5-82.0) years; their characteristics are shown in Supplementary Table S2. Written informed consent was obtained for all healthy individuals.

***ddPCR assessment of MN1/ABL1 copy numbers***

Mononuclear cells were isolated from peripheral blood. RNA was extracted from 1x10^7^ cells and processed to complementary DNA as previously described.^38^ Absolute *MN1* copy numbers were assessed using a probe based ddPCR assay (BioRad, Hercules, California, USA; Assay ID: dHsaCPE5040386) according to manufacturer’s specifications. Absolute *ABL1* copy numbers were assessed as previously described.^14^ ddPCR was performed on a QX100 platform (BioRad) and QuantaSoft software (Biorad) was used for raw data processing. With the droplet generator, each sample was divided into approximately 10,000 - 20,000 partitions (droplets). After PCR amplification the samples were placed into the droplet reader, where each droplet was read as positive or negative for the gene expression by issuing specific fluorescence signals (FAM and HEX). Redistribution according to the Poisson’s algorithm determined the absolute target copy number in the original sample.

***MN1*/*ABL1* cut-off definition**

Using the R package ‘OptimalCutpoints’^39^ the optimal cut-off of 0.2992 absolute pre-HSCT *MN1*/*ABL1* copies (high *vs* low) was determined to differentiate according to their relapse probability. To evaluate whether *MN1*/*ABL1* quantification in patients with very high pre-HSCT *MN1*/*ABL1* copy number allowed the identification of a very high risk group, a second optimal cut-off of 0.8693 absolute *MN1*/*ABL1* copy number was assessed in these patients and discriminated a cohort with higher (n=27, 69% of patients with high pre-HSCT *MN1*/*ABL1* copy numbers) or the topmost pre-HSCT *MN1*/*ABL1* copy numbers (n=12, 31% of patients with high pre-HSCT *MN1*/*ABL1* copy numbers).

***Flow cytometry, cytogenetics, and molecular markers***

In patients with pre-treatment bone marrow material available, cytogenetic analyses were performed centrally in our institution using standard banding techniques. In cases were no metaphases could be obtained, fluorescence *in-situ* hybridization (FISH) was used to screen for recurrent abnormalities (*i.e.* del5/5q, del7/7q, trisomy 8, abn11q23, t(8;21), inv(16) and t(15;17); [n=5]). At diagnosis, the presence of internal tandem duplication in the *FLT3* gene (*FLT3*-ITD), mutations in the *FLT3* tyrosine kinase domain (*FLT3*-TKD) and in the *NPM1* and *CEBPA* genes were determined as previously described.^40^ Patients were grouped according to the ELN 2010 classification in four risk groups.^41^ For 71 patients with material available, the bone marrow CD34 and CD38 expression on mononuclear cells at diagnosis was determined as previously described.^42^ Patients were considered CD34-positive when more than 20% of blasts at diagnosis reacted with the CD34 antibody.^43^

***Analysis of other MRD markers***

For all patients, pre-HSCT *BAALC*/*ABL1* copy numbers were evaluated by ddPCR as previously described.^14^ In 111 patients, *WT1*/*ABL1* expression levels prior to HSCT were evaluated using quantitative PCR as previously described.^13^ For 20 patients with *NPM1* mutated AML, pre-HSCT *NPM1* MRD status was evaluated by ddPCR as previously described.^8^ The applicability of all three markers for MRD evaluation have been previously published by our institution.^8,13,14^

***Definition of clinical endpoints and statistical analyses***

All statistical analyses were performed using the R statistical software platform (version 3.4.3). CIR was calculated from HSCT to morphologic relapse and OS was calculated from HSCT to death from any cause. Associations of the pre-HSCT *MN1*/*ABL1* copy numbers with baseline clinical, demographic, and molecular features were compared using the Kruskal-Wallis test and Fisher’s exact test for continuous and categorical variables, respectively. For OS, survival estimates were calculated using the Kaplan-Meier method and groups were compared with the log-rank test. CIR was calculated considering the competing risk NRM using the Fine and Gray model.

**Acknowledgements**

This study was supported by the Deutsche José Carreras Stiftung e.V. (04R/2016 and PS15/15), Verein Zusammen gegen den Krebs e.V., and Ein Herz für Kinder e.V.. The authors thank Christel Müller, Daniela Bretschneider, Evelin Hennig, Dr. Sabine Leiblein, Martina Pleß, Ulrike Bergmann, Janet Bogardt, Annette Jilo, and Dagmar Cron for their help in determing cytogenetic, morphologic and immunological analyses; Christine Günther, Scarlett Schwabe, Ines Kovacs, and Kathrin Wildenberger for their help in sample processing and Dr. Max Hubmann and Prof. Ralf Burckhardt in for their help in *WT1* expression analysis..

**Conflicts-of-Interest disclosure**

The authors declare no conflict of interest.

**References**

**1.** Cancer Genome Atlas Research Network. Genomic and epigenomic landscapes of adult de novo acute myeloid leukemia. *N Engl J Med*. 2013;368(22):2059-74.

**2.** Döhner H, Estey E, Grimwade D, Amadori S, Appelbaum FR, Büchner T, et al. Diagnosis and management of AML in adults: 2017 ELN recommendations from an international expert panel. *Blood.* 2017;129(4):424-447.

**3.** Döhner H, Weisdorf DJ, Bloomfield CD. Acute Myeloid Leukemia. *N Engl J Med*. 2015;373(12):1136-52.

**4**. Ivey A, Hills RK, Simpson MA, Jovanovic JV, Gilkes A, Grech A, et al. Assessment of Minimal Residual Disease in Standard-Risk AML. *N Engl J Med*. 2016;374(5):422-33.

**5.** Hourigan CS, Gale RP, Gormley NJ, Ossenkoppele GJ, Walter RB. Measurable residual disease testing in acute myeloid leukaemia. *Leukemia*. 2017;31(7):1482-90.

**6.** Grimwade D, Freeman SD. Defining minimal residual disease in acute myeloid leukemia: which platforms are ready for "prime time"? *Blood*. 2014;124(23):3345-55.

**7.** Doubek M, Palasek I, Pospisil Z, Borsky M, Klabusay M, Brychtova Y, et al. Detection and treatment of molecular relapse in acute myeloid leukemia with *RUNX1* (AML1), *CBFB*, or *MLL* gene translocations: frequent quantitative monitoring of molecular markers in different compartments and correlation with *WT1* gene expression. *Exp Hematol*. 2009;37(6):659-72.

**8.** Bill M, Grimm J, Jentzsch M, Kloss L, Goldmann K, Schulz J, et al. Digital droplet PCR-based absolute quantification of pre-transplant *NPM1* mutation burden predicts relapse in acute myeloid leukemia patients. *Ann Hematol*. 2018;97(10):1757-65.

**9**. Ommen HB, Schnittger S, Jovanovic JV, Ommen IB, Hasle H, Østergaard M, et al. Strikingly different molecular relapse kinetics in *NPM1c*, *PML-RARA*, *RUNX1-RUNX1T1*, and *CBFB-MYH11* acute myeloid leukemias. *Blood.* 2010;115(2):198-205.

**10.** Ding L, Ley TJ, Larson DE, Miller CA, Koboldt DC, Welch JS, et al. Clonal evolution in relapsed acute myeloid leukaemia revealed by whole-genome sequencing. *Nature*. 2012;481(7382):506-10.

**11**. Grimwade D, Ivey A, Huntly BJ. Molecular landscape of acute myeloid leukemia in younger adults and its clinical relevance. *Blood*. 2016;127(1):29-41.

**12.** Ostergaard M, Olesen LH, Hasle H, Kjeldsen E, Hokland P. *WT1* gene expression: an excellent tool for monitoring minimal residual disease in 70% of acute myeloid leukaemia patients - results from a single-centre study. *Br J Haematol.* 2004;125(5):590–600.

**13.** Lange T, Hubmann M, Burkhardt R, Franke GN, Cross M, Scholz M, et al. Monitoring of *WT1* expression in PB and CD34(+) donor chimerism of BM predicts early relapse in AML and MDS patients after hematopoietic cell transplantation with reduced-intensity conditioning*. Leukemia*. 2011;25(3):498-505.

**14.** Jentzsch M, Bill M, Grimm J, Schulz J, Goldmann K, Beinicke S, et al. High *BAALC* copy numbers in peripheral blood prior to allogeneic transplantation predict early relapse in acute myeloid leukemia patients. *Oncotarget*. 2017;8(50):87944-54.

**15**. Weber S, Haferlach T, Alpermann T, Perglerová K, Schnittger S, Haferlach C, et al. Feasibility of *BAALC* gene expression for detection of minimal residual disease and risk stratification in normal karyotype acute myeloid leukaemia. *Br J Haematol*. 2016;175(5):904-16.

**16**. Najima Y, Ohashi K, Kawamura M, Onozuka Y, Yamaguchi T, Akiyama H, et al. Molecular monitoring of *BAALC* expression in patients with CD34-positive acute leukemia. *Int J Hematol*. 2010;91(4):636–45.

**17.** Heuser M, Beutel G, Krauter J, Döhner K, von Neuhoff N, Schlegelberger B, et al. High meningioma 1 (*MN1*) expression as a predictor for poor outcome in acute myeloid leukemia with normal cytogenetics. *Blood*. 2006;108(12):3898-905.

**18.** Carella C, Bonten J, Sirma S, Kranenburg TA, Terranova S, Klein-Geltink R, et al. *MN1* overexpression is an important step in the development of inv(16) AML*. Leukemia*. 2007;21(8):1679-90.

**19.** Heuser M, Argiropoulos B, Kuchenbauer F, Yung E, Piper J, Fung S, et al. *MN1* overexpression induces acute myeloid leukemia in mice and predicts ATRA resistance in patients with AML. *Blood.* 2007;110(5):1639-47.

**20.** Schwind S, Marcucci G, Kohlschmidt J, Radmacher MD, Mrózek K, Maharry K, et al. Low expression of *MN1* associates with better treatment response in older patients with de novo cytogenetically normal acute myeloid leukemia. *Blood.* 2011;118(15):4188-98.

**21**. Langer C, Marcucci G, Holland KB, Radmacher MD, Maharry K, Paschka P, et al. Prognostic importance of *MN1* transcript levels, and biologic insights from MN1-associated gene and microRNA expression signatures in cytogenetically normal acute myeloid leukemia: a cancer and leukemia group B study. *J Clin Oncol*. 2009;27(19):3198-204.

**22.** Carturan S, Petiti J, Rosso V, Calabrese C, Signorino E, Bot-Sartor G, et al. Variable but consistent pattern of Meningioma 1 gene (*MN1*) expression in different genetic subsets of acute myelogenous leukaemia and its potential use as a marker for minimal residual disease detection. *Oncotarget*. 2016;7(45):74082-96.

**23.** Gupta V, Tallman MS, Weisdorf DJ. Allogeneic hematopoietic cell transplantation for adults with acute myeloid leukemia: myths, controversies, and unknowns. *Blood.* 2011;117(26):2307-18.

**24.** McSweeney PA, Niederwieser D, Shizuru JA, Sandmaier BM, Molina AJ, Maloney DG, et al. Hematopoietic cell transplantation in older patients with hematologic malignancies: replacing high-dose cytotoxic therapy with graft-versus-tumor effects. *Blood*. 2001;97(11):3390-400.

**25.** Araki D, Wood BL, Othus M, Radich JP, Halpern AB, Zhou Y, et al. Allogeneic Hematopoietic Cell Transplantation for Acute Myeloid Leukemia: Time to Move Toward a Minimal Residual Disease-Based Definition of Complete Remission? *J Clin Oncol*. 2016;34(4):329-36.

**26.** Muffly L, Pasquini MC, Martens M, Brazauskas R, Zhu X, Adekola K, et al. Increasing use of allogeneic hematopoietic cell transplantation in patients age 70 years and older in the United States. *Blood*. 2017;130(9):1156-64.

**27.** Horowitz MM, Gale RP, Sondel PM, Goldman JM, Kersey J, Kolb HJ, et al. Graft-versus-leukemia reactions after bone marrow transplantation. *Blood.* 1990;75(3):555-62.

**28.** Hindson CM, Chevillet JR, Briggs HA, Gallichotte EN, Ruf IK, Hindson BJ, et al. Absolute quantification by droplet digital PCR versus analog real-time PCR. *Nat Methods*. 2013;10(10):1003-5.

**29.** Venditti A, Piciocchi A, Candoni A, Melillo L, Calafiore V, Cairoli R, et al. Risk-adapted, MRD-directed therapy for young adults with newly diagnosed acute myeloid leukemia: results of the AML1310 trial of the GIMEMA group [abstract]. *Haematologica*. 2017. Abstract S111.

**30.** Kawagoe H, Grosveld GC. MN1-TEL myeloid oncoprotein expressed in multipotent progenitors perturbs both myeloid and lymphoid growth and causes T-lymphoid tumors in mice. *Blood*. 2005;106(13):4278-86.

**31.** Valk PJ, Verhaak RG, Beijen MA, Erpelinck CA, Barjesteh van Waalwijk van Doorn-Khosrovani S, Boer JM, et al. Prognostically useful gene-expression profiles in acute myeloid leukemia. *N Engl J Med*. 2004;350(16):1617-28.

**32.** Guolo F, Minetto P, Clavio M, Miglino M, Galaverna F, Raiola AM, et al. Combining flow cytometry and *WT1* assessment improves the prognostic value of pre-transplant minimal residual disease in acute myeloid leukemia. *Haematologica*. 2017;102(9):e348-e351.

**33.** Heuser M, Wingen LU, Steinemann D, Cario G, von Neuhoff N, Tauscher M, et al. Gene-expression profiles and their association with drug resistance in adult acute myeloid leukemia. *Haematologica.* 2005;90(11):1484-92.

**34.** Zeijlemaker W, Meijer R, Kelder A, Carbaat-Ham J, Oussoren-Brockhoff Y, Snel S, et al. Leukemic stem cell frequency combined with MRD is an important biomarker to predict relapse in acute myeloid leukemia. Results from a prospective H102 study. [abstract]. *Haematologica*. 2017. Abstract S113.

**35**. Falini B, Mecucci C, Tiacci E, Alcalay M, Rosati R, Pasqualucci L, et al. Cytoplasmic nucleophosmin in acute myelogenous leukemia with a normal karyotype. *N Engl J Med*. 2005;352(3):254-66.

**36.** Getta BM, Devlin SM, Levine RL, Arcila ME, Mohanty AS, Zehir A, et al. Multicolor Flow Cytometry and Multigene Next-Generation Sequencing Are Complementary and Highly Predictive for Relapse in Acute Myeloid Leukemia after Allogeneic Transplantation. *Biol Blood Marrow Transplant*. 2017;23(7):1064-71.

**37.** Hegenbart U, Niederwieser D, Sandmaier BM, Maris MB, Shizuru JA, Greinix H, et al. Treatment for acute myelogenous leukemia by low-dose, total-body, irradiation-based conditioning and hematopoietic cell transplantation from related and unrelated donors. *J Clin Oncol*. 2006;24(3):444-53.

**38.** Lange T, Niederwieser DW, Deininger MW. Residual disease in chronic myeloid leukemia after induction of molecular remission. *N Engl J Med.* 2003; 349(15):1483-84.

**39.** López-Ratón M, Rodríguez-Álvarez M, Cadarso-Suárez C, Gude-Sampedro F. OptimalCutpoints: An R Package for Selecting Optimal Cutpoints in Diagnostic Tests. *J Stat Software.* 2014; Volume 61, Issue 8.

**40.** Bill, M, Jentzsch, M, Grimm, J, Schubert K, Lange T, Cross M, et al. Prognostic impact of the European LeukemiaNet standardized reporting system in older AML patients receiving stem cell transplantation after non-myeloablative conditioning. *Bone Marrow Transplantation*. 2017;52(6):932-35.

**41.** Döhner H, Estey EH, Amadori S, Appelbaum FR, Büchner T, Burnett AK, et al. Diagnosis and management of acute myeloid leukemia in adults: recommendations from an international expert panel, on behalf of the European LeukemiaNet. *Blood*. 2010;115(3):453-74.

**42.** Jentzsch M, Bill M, Nicolet D, Leiblein S, Schubert K, Pless M, et al. Prognostic Impact of the CD34+/CD38- Cell Burden in Patients with Acute Myeloid Leukemia receiving Allogeneic Stem Cell Transplantation. *Am J Hematol*. 2017;92(4):388-96.

**43.** Bene MC, Castoldi G, Knapp W, Ludwig WD, Matutes E, Orfao A et al. Proposals for the immunological classification of acute leukemias. European Group for the Immunological Characterization of Leukemias (EGIL). *Leukemia.* 1995; 9(10): 1783–6.

**Figure Legends**

**Figure 1. Comparison of pre-HSCT *MN1*/*ABL1* copy numbers in AML patients (n=124) and healthy controls (n=17).**

**Figure 2.** **Time from HSCT to relapse according to high (median 70, range 20-363) or low (median 124, range 19-543) pre-HSCT *MN1*/*ABL1* copy numbers, 0.30 cut, in patients suffering relapse after HSCT (n=45).**

**Figure 3.** **Outcome according to pre-HSCT *MN1*/*ABL1* copy numbers for the whole cohort (n=124).** According to high *vs*. low, 0.30 cut: (A) Cumulative Incidence of Relapse and (B) Overall Survival; and according to the topmost *vs*. higher *vs*. low, 0.87 and 0.30 cut: (C) Cumulative Incidence of Relapse and (D) Overall Survival.

**Figure 4. Outcome according to pre-HSCT *MN1*/*ABL1* copy numbers, high *vs*. low, 0.30 cut, in mutated *NPM1* MRD^neg^ patients (n=15).** (A) Cumulative Incidence of Relapse and (B) Overall Survival.
